# Personality Inference From Semistructured Interviews Using Large Language Models: Comparative Evaluation Study

**DOI:** 10.2196/87129

**Published:** 2026-10-16

**Authors:** Jianfeng Zhu, Julina Maharjan, Xinyu Li, Karin G Coifman, Ruoming Jin

**Affiliations:** 1 Department of Computer Science, Kent State University, 241 Mathematical Sciences Building, Kent, OH, 44242, United States, 1 3306729107; 2 Department of Psychological Sciences, Kent State University, Kent, OH, United States

**Keywords:** large language models, LLMs, personality inference, Big Five personality traits, semistructured interviews, low-rank adaptation, LoRA fine-tuning

## Abstract

**Background:**

Personality traits are central to understanding human behavior and life outcomes, yet their assessment remains challenging due to biases and limited expressiveness in traditional self-report inventories. The emergence of large language models (LLMs) creates new opportunities to infer personality from naturalistic text, but it remains unclear whether these systems can reliably predict validated personality traits or whether their predictions primarily reflect superficial linguistic patterns.

**Objective:**

This study evaluates the ability of LLM-based methods to predict continuous Big Five personality scores (1‐5 Likert scale) from semistructured interview transcripts and compares the performance of different modeling paradigms.

**Methods:**

We analyzed semistructured interview transcripts from 518 participants, each paired with validated self-reported Big Five scores measured using the 10-item Big Five Inventory (BFI-10). We compared four paradigms: (1) zero-shot and chain-of-thought prompting with GPT-5 mini; (2) parameter-efficient fine-tuning (low-rank adaptation) applied to open-source RoBERTa-base and LLaMA-2-7B models; (3) supervised fine-tuning of GPT-4.1 mini across two independent training runs; and (4) embedding-based regression using 4 pretrained encoders (all-mpnet-base-v2, E5-large-v2, text-embedding-3-small, and text-embedding-3-large) with a multioutput ridge regression.

**Results:**

Supervised fine-tuning yielded the strongest overall performance among the evaluated approaches. In the best-performing run, correlations reached *r*=0.718 for conscientiousness and *r*=0.631 for agreeableness. Neuroticism showed moderate and consistent performance across the 2 fine-tuning runs, whereas extraversion was less stable across runs. Embedding-based regression, particularly with text-embedding-3-large, achieved competitive results for conscientiousness and agreeableness, although it remained below supervised fine-tuning. Prompt-based methods showed weak and inconsistent performance across traits. Openness was generally difficult to predict, although RoBERTa-base with embedding-based low-rank adaptation fine-tuning showed modest but statistically significant performance, indicating trait- and method-dependent variability.

**Conclusions:**

LLM-based approaches exhibit trait-dependent performance in predicting personality from naturalistic dialogue, with fine-tuning substantially improving correspondence with validated psychometric scores. These findings suggest that personality inference from naturalistic dialogue is feasible but depends strongly on modeling strategy, trait domain, and the availability of supervised fine-tuning with validated personality measures.

## Introduction

### Background and Study Rationale

Personality traits play a fundamental role in understanding individual differences, social behavior, and life outcomes [1-3]. However, their assessment remains challenging, as traditional self-report inventories are subject to response biases and may be limited in their ability to capture the richness of individual expression [4,5]. Prior research suggests that personality-relevant information is reflected in everyday language use, as individuals express stable patterns of thought and behavior in how they speak and write [6-8]. This perspective is grounded in the lexical hypothesis, which posits that meaningful aspects of personality are encoded in natural language and expressed through everyday communication [9-11].

Building on this foundation, recent advances in large language models (LLMs) have enabled personality prediction directly from text, offering scalable and context-sensitive alternatives to traditional assessment methods [12-18]. Existing studies suggest that LLMs can infer Big Five traits from language with modest accuracy, primarily using social media data or written corpora [19-21]. Approaches leveraging extended conversational data, such as interview transcripts, have shown improved performance, suggesting that richer, context-aware language better captures personality-relevant signals [16,22-24].

However, existing work remains limited in 3 key respects. First, many studies frame personality as categorical labels rather than continuous traits, limiting psychometric fidelity [14,25]. Second, most datasets rely on social media text [26-29], which may not reflect structured or task-oriented conversational contexts. Third, few studies jointly integrate ecologically grounded interview data [30], validated individual-level trait measures, and systematic comparisons of multiple LLM modeling paradigms under a unified evaluation framework. As a result, it remains unclear how well LLMs can predict personality from interview data in ways that align with established psychometric standards. Our prior research began to explore this challenge using prompt-based inference on the same interview-based benchmark [24], and the present study extends that work by incorporating supervised fine-tuning and representation-based regression approaches.

The Big Five, or Five-Factor Model, is the most widely accepted framework in personality psychology [31], comprising 5 broad trait dimensions: openness, conscientiousness, agreeableness, extraversion, and neuroticism. To address the gaps identified above, this study evaluates whether contemporary LLMs can predict continuous Big Five personality scores from semistructured interview transcripts paired with validated 10-item Big Five Inventory (BFI-10) self-report scores. This design better approximates naturalistic human communication and enables a more realistic assessment of model performance against psychometrically grounded benchmarks.

We compare 4 complementary modeling paradigms reflecting distinct assumptions about how personality signals are encoded and extracted from text. First, we examine prompt-based inference using GPT-5 mini (OpenAI) under both zero-shot and chain-of-thought (CoT) settings, representing inference without task-specific adaptation. Second, we evaluate parameter-efficient fine-tuning via low-rank adaptation (LoRA) applied to RoBERTa-base (Robustly Optimized BERT Approach; Meta AI) and LLaMA-2-7B (Large Language Model Meta AI; Meta Platforms, Inc). Third, we investigate supervised model adaptation through supervised fine-tuning of GPT-4.1 mini (OpenAI), which we evaluate across 2 independent training runs. Fourth, we assess embedding-based regression, in which fixed representations from 4 pretrained encoders (all-mpnet-base-v2 [Microsoft], E5-large-v2 [Microsoft], text-embedding-3-small [OpenAI], and text-embedding-3-large [OpenAI]) are used to predict personality traits via a multioutput ridge regression model. By contrasting these paradigms, we aim to disentangle the contributions of prompting strategy, representation learning, and different levels of model adaptation in personality inference and to identify which traits are most reliably captured from naturalistic conversational data.

### Related Work

#### Traditional Text-Based Personality Recognition

Automatic personality recognition has long been studied in natural language processing. Early approaches relied on classical machine learning techniques (eg, support vector machines and regression) [32], combined with handcrafted linguistic features derived from resources such as linguistic inquiry and word count categories [33], emotion lexicons [34], and term frequency-inverse gravity moment [35]. Applied to essays, blogs, and social media text, these methods demonstrated that stable personality signals are present in written language [36]. Meta-analytic evidence supports this pattern, although effect sizes typically remain modest and vary considerably across traits, platforms, and modeling choices [37,38].

Subsequent advances introduced transformer-based models such as BERT (bidirectional encoder representations from transformers) [39,40], which leverage large-scale pretraining and fine-tuning on datasets such as myPersonality [41] and PANDORA [42]. These models improved predictive accuracy but continued to rely on binary or categorical labels and nonclinical data, limiting their psychometric fidelity and generalizability. These limitations have motivated the exploration of more flexible, language-driven approaches based on LLMs.

#### Data Paradigms: Social Media, Author Profiling, and Conversational Data

Much of the existing work in personality recognition is grounded in social media and author profiling datasets. Shared tasks such as the PAN 2015 Author Profiling task and datasets such as PANDORA have provided valuable scale for personality computing, but they are largely grounded in tweets or Reddit posts rather than elicited, topic-diverse narratives [25,28,29,41,43,44]. However, these datasets are primarily based on short-form text (eg, tweets or Reddit posts), which may not reflect how individuals express themselves in structured or context-rich settings.

In parallel, persona-conditioned dialogue resources such as Persona-Chat [45] have been developed to support controllable text generation conditioned on predefined personality profiles. While useful for generation tasks, such datasets do not capture validated trait scores from real individuals and, therefore, are not directly suitable for psychometric evaluation.

#### LLM-Based Personality Inference and Modeling Paradigms

Recent studies have examined LLMs for personality-related language tasks, ranging from personality-conditioned dialogue generation to personality trait inference. Prompt-based methods, including zero-shot, few-shot prompting, and CoT prompting, provide lightweight strategies for eliciting personality-related judgments without task-specific fine-tuning [24,46-48]. Peters and Matz [29] reported that GPT-3.5 and GPT-4 achieved moderate correlations with self-reported traits using zero-shot prompting on Facebook (Meta Platforms, Inc) status updates. Similarly, Piastra and Catellani [19] found small-to-moderate predictive performance when applying ChatGPT (OpenAI) to written text. More structured approaches, such as PsyCoT, use psychological questionnaire items as explicit reasoning steps and have been shown to improve GPT-3.5 (OpenAI) performance on personality detection benchmarks, although these gains have primarily been demonstrated in classification settings rather than in continuous trait prediction [49].

More substantial progress has been achieved through approaches that incorporate supervised learning and parameter adaptation. Embedding-based methods, which use LLM-derived representations as inputs to downstream models, have been shown to outperform prompt-based inference in several settings [25]. Parameter-efficient fine-tuning techniques, such as LoRA [50], further improve adaptation while reducing computational cost, enabling scalable personality prediction across domains [51]. Recent frameworks such as PersLLM [52] extend this idea by combining LLM-based representation learning with lightweight output networks and memory layers, reducing redundant computation while maintaining competitive performance. In parallel, retrieval-augmented methods, including retrieval-augmented generation (RAG) [53] and PersonaRAG [54], introduce mechanisms for grounding predictions in external or user-specific data, highlighting the potential of personalized and context-aware inference.

Table 1 summarizes major LLM-based personality recognition studies published between 2021 and 2025, highlighting rapid methodological innovation alongside persistent challenges in data quality, labeling schemes, and interpretability.

**Table 1.** Comparative overview of recent large language model (LLM)–based personality recognition studies published between 2021 and 2025.

Study	LLM(s) used	Methodology	Dataset	Label type	Key finding/limitation
Christian et al [55], 2021	BERT, RoBERTa, and XLNet	Used a multimodel deep learning architecture using pretrained language models as feature extractors, combined with model averaging	myPersonality	Binary	Outperformed previous works by combining multiple data sources and models, achieving high classification accuracy (eg, 88.5% on Twitter [subsequently rebranded X] data)
Ji et al [56], 2023	ChatGPT	Used prompting strategies (zero-shot, one-shot, and CoT^a^); zero-shot CoT most effective for explanation and prediction	Essays and PANDORA	Binary	CoT improves over basic zero-shot but still trails fine-tuned state-of-the-art models
Sirasapalli and Malla [57], 2023	BERT	Proposed data fusion, mapping MBTI^b^ dataset to Big Five traits, and combining with other corpora	Essays, myPersonality, and MBTI	Binary	Data fusion increased labeled data and model performance, reaching 87.9% accuracy
Serapio-García et al [15], 2025	PaLM family (18 LLMs)	Assessed LLM personality using validated psychometric tests (IPIP-NEO^c^ and BFI^d^) under various prompting	Psychometric Tests	N/A^e^ (assessment)	Simulated LLM personality was reliable and valid; shaped via prompting
Hu et al [58], 2024	LLM (unspecified)	Used LLMs to generate augmentations for contrastive distillation	Kaggle personalitycafe and PANDORA	Binary	LLM-augmented data improves task-specific models
Peters and Matz [29], 2024	GPT-4, LLaMA 2, and Mistral	Used zero-shot and CoT for continuous prediction; assessed with correlation and error	Essays	Continuous	Weak correlation (*r*<0.30); minimal gain from CoT
Wang et al [59], 2025	GPT-4	Emulated individuals using personality scores; completed questionnaires as that in-person	Human personality data	Continuous	Emulated responses had high convergent validity (*r*>0.90) but superior (less realistic) consistency
Maharjan et al [25], 2025	RoBERTa, BERT, and OpenAI	Generated LLM embeddings and trained bidirectional long short-term memory model for continuous scores	PANDORA	Continuous	Embedding models outperform zero-shot
This work	GPT-4.1 mini, GPT-5 mini, LLaMA-2-7B, and RoBERTa-base	Compares zero-shot and CoT prompting, LoRA^f^ fine-tuning, supervised fine-tuning, and embedding-based regression on interview transcripts, with validated Big Five scores	Real-world interviews	Continuous	Provide a systematic comparison of 4 LLM paradigms on ecologically grounded interview data with validated psychometric labels; fine-tuning substantially outperforms prompting

a CoT: chain-of-thought.

b MBTI: Myers-Briggs Type Indicator.

c IPIP-NEO: International Personality Item Pool–neuroticism, extraversion, and openness.

d BFI: Big Five Inventory.

e N/A: not applicable.

f LoRA: low-rank adaptation.

Collectively, prior work highlights both the promise and the fragmentation of LLM-based personality inference, with substantial variation in data sources, labeling schemes, and modeling paradigms. Our prior work evaluated LLMs (eg, GPT-5 mini, GPT-4.1 mini, Meta-LLaMA [Meta Platforms, Inc], and DeepSeek) using zero-shot and CoT prompting on the same interview-based benchmark and observed consistently weak performance in personality prediction [24].

These findings suggest that limitations arise not only from model capacity but also from reliance on prompt-based inference. The present study moves beyond prompt-based inference by introducing supervised fine-tuning and representation-based approaches, including model fine-tuning and embedding-based regression, to enable explicit learning from labeled data.

### Dataset and Participants

In this study, we used real-world, semistructured interview transcripts from a pooled dataset of 555 adults across 5 prior investigations of adjustment to stress. The pooled source dataset included active-duty firefighters (n=103), patients with multiple sclerosis (n=61), adults recruited following injury or traumatic events (n=254), first-year college students (n=84), and community adults (n=53). Of the 555 participants in the pooled source dataset, 518 had complete BFI-10 responses and were included in the present analysis. The mean scores for the Big Five traits are as follows: openness (3.35, SD 0.63), extraversion (3.03, SD 1.09), neuroticism (2.93, SD 1.23), conscientiousness (2.74, SD 1.30), and agreeableness (2.63, SD 1.13), indicating that average trait levels were near the midpoint of the scale. Full descriptive statistics are reported in Table A7 of Multimedia Appendix 1.

The analytic sample was demographically diverse and balanced by sex (n=260 male and n=257 female participants), had minimal missing data, and spanned a wide age range (mean 39.32, SD 16.45). Participants represented various racial backgrounds (n=402 White and n=116 non-White/Other participants) and educational levels ranging from high school to college and above. Additional details regarding cohort characteristics and interview prompts are available via the Open Science Framework (OSF; Center for Open Science) repository [60]. The complete semistructured interview prompts are provided in Table A1 of Multimedia Appendix 1. All code used in this study is publicly available at the project repository [61].

The semistructured interview protocol varied across the 5 parent studies to reflect the specific context and circumstances of each sample. Depending on the parent study, participants completed 3 to 5 prompts, beginning with a neutral description of previous-day activities and followed by study-specific prompts concerning relevant experiences, coping, and/or positive and negative events. Participants were encouraged to speak spontaneously for up to 3 minutes in response to each prompt [62-64]. Interviews were audio-recorded and transcribed using established conventions. The resulting transcripts showed substantial variability in length. On average, participants produced 2955 (SD 1855) words per interview, with a range from 387 to 7143 words (median 2292, IQR 1569‐4388). Representative excerpts from 1 participant in the active-duty firefighter parent study are presented in Table 2.

**Table 2.** Representative responses from a semistructured experiential interview with 1 participant from the active-duty firefighter parent study, selected from the pooled analytic sample (N=518).

Administered prompt	Response excerpt
Baseline: previous-day activities	“Beginning of the day... I have two sons... played outside... gym...”
Q1: experience of being a firefighter	“Being a firefighter... challenging and amazing experience... bad experiences too...”
Q2: coping with firefighter work	“You talk to people you trust at work... my wife and I have been married...”
Q3: scary or frightening event	“First big incident... stressful leading into it...”
Q4: positive firefighter event	“First baby we delivered—early morning call... heroin case...”

## Methods

### Prompt-Based Inference

To evaluate the capacity of LLMs for personality inference, we used the GPT-5 mini via the OpenAI API (model ID: *gpt-5-mini-2025-08-07;* [65]) to predict Big Five trait scores from naturalistic interview transcripts. The model was instructed to act as a psychologically insightful agent and to generate continuous scores between 1.0 and 5.0 for each of the five traits.

We implemented 2 prompting strategies. The first was a standard zero-shot prompt that directly instructed the model to return trait scores based on the interview content [66]. The second was a CoT prompt that encouraged intermediate reasoning about emotional tone and linguistic markers prior to scoring [67]. For prompt-based inference, transcripts were used in their conversational form, with interviewer prompts preserved to retain contextual structure. Both prompting strategies were executed using the same API configuration without specifying a temperature parameter. Model outputs followed a predefined score format, and trait scores were automatically extracted using rule-based parsing implemented with regular expressions. Compared with our prior work [24], the prompts were slightly refined with additional instructions while maintaining the same overall prompting structure. The complete zero-shot and CoT prompt templates, along with an example model output, are provided in the Textboxes 1 to 3 of Multimedia Appendix 1.

### LoRA-Based Adaptation

To improve prediction performance with respect to self-reported Big Five personality scores, we applied LoRA [50] for efficient fine-tuning of transformer-based language models. Here, r denotes the rank of the low-rank decomposition, representing the dimensionality of the trainable adaptation matrices. A larger rank increases model capacity but requires more parameters, whereas a smaller rank improves parameter efficiency. Specifically, we fine-tuned the open-source LLaMA-2-7B model [68] to jointly predict all 5 personality traits from the input text. LoRA modules are injected into the attention and feedforward layers, allowing for parameter-efficient adaptation while preserving the model’s general language capabilities.

Our implementation used LoRA fine-tuning with 4-bit quantization to reduce memory requirements. The training configuration used a learning rate of 1e-4, a per-device batch size of 2, gradient accumulation steps of 4, weight decay of 0.01, and 3 training epochs. The LoRA configuration used a scaling factor α of 64, a dropout rate of 0.05, and no bias terms. In the rank-sensitivity experiments, adapter ranks of 8, 16, and 32 were evaluated using the same configuration unless otherwise specified.

In addition to decoder-based models, we evaluated encoder-only architectures to assess the role of representation learning in personality prediction. We used RoBERTa-base [39] as a representative encoder model. Because of its limited input length, interview transcripts were tokenized and segmented into nonoverlapping 512-token chunks, with an overlap ratio of 0.0 and a stride of 512 tokens. Each chunk was encoded independently, and chunk-level embeddings were aggregated using mean pooling to form a global transcript representation. A lightweight nonlinear regression head was then trained on these pooled embeddings to predict continuous trait scores.

### Supervised Fine-Tuning of GPT-4.1 mini

To examine whether supervised model adaptation improves personality trait prediction, we conducted supervised fine-tuning using a pretrained, closed-source LLM. Specifically, we used GPT-4.1 mini (April 14, 2025; [69]) as the base model and trained it on the personality prediction dataset.

Each training instance consisted of a participant’s textual responses as the input and the corresponding continuous scores for the 5 Big Five personality traits as the target outputs. The dataset was split into an 80% fine-tuning set and a 20% test set. Fine-tuning was performed using a supervised learning objective for 3 epochs, with a batch size of 1 and a learning rate multiplier of 2. No separate validation set was used.

To assess the consistency of the fine-tuning process, we conducted 2 independent training runs using the same hyperparameter configuration. These runs resulted in 2 models (FT1 and FT2), which were evaluated separately.

### Embedding-Based Regression

To complement prompt- and fine-tuning–based approaches, we evaluated static embedding-based personality inference across 4 pretrained encoders of varying scales and training paradigms: all-mpnet-base-v2 (768-dimensional, built on masked and permuted pretraining for language understanding) [70] from the Sentence-Transformers library; E5-large-v2 (1,024-dimensional) [71], trained with contrastive learning on large-scale text pairs and requiring a “*passage*” (prefix at inference time); and OpenAI text-embedding-3-small (1536-dimensional) and text-embedding-3-large (3072-dimensional), accessed via the OpenAI API [72]. This selection spans a broad range of model capacities and training paradigms, enabling systematic comparison among open-source encoders, contrastive-learning–based embeddings, and large-scale commercial models.

For all models, each participant’s interview transcript was encoded into a fixed-size dense vector at the transcript level. These embeddings, standardized to have 0 mean and unit variance, were passed to a multioutput ridge regression model (α=1.0) [73] that jointly predicted all 5 Big Five trait scores. The dataset was split into 80% training and 20% testing partitions. We further conducted a sample-size sensitivity analysis by training the text-embedding-3-small ridge regression model on increasing proportions of the training pool (20%, 40%, 60%, 80%, and 100%) and evaluating performance on the same held-out test set. The 20% to 80% conditions were repeated 5 times with different random subsamples; the 100% condition used the full training pool.

### Evaluation Metrics

We assessed model performance using 2 complementary metrics: Pearson correlation (*r*) and mean absolute error (MAE). Pearson correlation quantifies the consistency between model-predicted and self-reported trait scores, serving as an indicator of criterion-related validity. Correlations above *r*=0.5 are typically interpreted as stronger evidence of convergence with self-report scores, indicating that the model captures meaningful individual differences in personality. Values between 0.3 and 0.5 suggest moderate agreement with self-report scores, while correlations below 0.3 suggest weak predictive consistency. MAE provides a direct measure of prediction error on the original trait scale (1.0‐5.0). Lower MAE values (eg, ≤0.5) reflect high precision, while values between 0.5 and 0.8 are common and may be acceptable depending on context. MAE values exceeding 1.0 typically signal poor calibration or unreliable estimates. Unlike correlation, MAE is sensitive to systematic bias and offers a more interpretable estimate of absolute prediction fidelity.

We also calculated sample-specific internal consistency for each-item trait scale and reported both Cronbach α and interitem correlations in Table A9 of Multimedia Appendix 1.

### Ethical Considerations

This study used deidentified semistructured interview transcripts and BFI-10 personality assessment data collected from 5 prior Kent State University Institutional Review Board–approved studies under protocol numbers #17‐056, #14‐233, #13‐134, #15‐446, and #12‐257 [60]. Participants provided informed consent in the original studies for the use of their data for research purposes. The present study involved secondary analysis of deidentified data and did not involve recontacting participants or collecting new identifiable information; therefore, additional informed consent was not required for this analysis.

To protect participant privacy and confidentiality, all textual and psychometric data were anonymized or deidentified before analysis and direct identifiers were not used in model training, evaluation, or reporting. Results are reported only in aggregate. Any interview excerpts included in the manuscript were shortened or edited to avoid revealing identifying details. No identifiable participant images, screenshots, or user-identifying information are included in the manuscript or supplementary materials. Participants in the original studies were financially compensated for their time at a rate of approximately US $10 per hour. The total compensation varied across the parent studies because study activities and participation requirements differed; study-specific procedures are described in the corresponding published reports [74-78].

Given the sensitive nature of personality prediction, the models evaluated in this study are intended for research purposes only. They should not be used to identify individuals, make diagnostic judgments, or support high-stakes decisions without appropriate validation, ethical oversight, and human review.

## Results

### Prompt-Based Inference

Table 3 summarizes the performance of GPT-5 mini using zero-shot and CoT prompting. Overall, prompt-based methods exhibited weak and inconsistent performance across traits. The highest correlation was observed for *conscientiousness* in the zero-shot setting (*r*=0.158). Most other traits, including *neuroticism*, *openness*, and *extraversion*, showed correlations near 0 or were negative, indicating limited predictive validity. Prediction errors remained high across all traits. Only openness achieved a MAE below 0.7, whereas MAE values for conscientiousness and agreeableness exceeded 1.4, indicating poor calibration of predicted trait scores.

**Table 3.** Performance of prompt-based GPT-5 mini in predicting continuous Big Five personality scores from semistructured interviews in the pooled analytic sample (N=518).

Trait	Zero-shot (*r*^a^)	CoT^b^ (*r*)
Openness	−0.034	−0.010
Conscientiousness	0.158^c^	0.155^c^
Extraversion	−0.092^c^	−0.092^c^
Agreeableness	0.067	0.099^c^
Neuroticism	−0.026	−0.034

a *r* denotes the Pearson correlation coefficient.

b CoT: chain-of-thought.

c Values are statistically significant at *P*<.05. Detailed statistical results, including *P* values and CIs, are reported in Table A2 of Multimedia Appendix 1.

### LoRA-Based Adaptation

LoRA-based fine-tuning substantially improved personality prediction performance compared to prompt-based methods, with effects varying across traits and adaptation ranks.

As shown in Table 4, performance gains were most pronounced for agreeableness, which showed statistically significant correlations across all configurations (*r*=0.299 at rank 8, *r*=0.329 at rank 16, and *r*=0.229 at rank 32). Openness showed a different pattern, with performance improving as rank increased and reaching statistical significance only at rank 32 (*r*=0.216, *P*<.05).

**Table 4.** Performance of LLaMA-2-7B low-rank adaptation (LoRA) fine-tuning across adapter ranks for predicting continuous Big Five personality scores from semistructured interviews in the pooled analytic sample (N=518).

Trait	Rank 8 (*r*^a^)	Rank 16 (*r*)	Rank 32 (*r*)
Openness	0.042	0.127	0.216^b^
Conscientiousness	−0.057	0.032	−0.055
Extraversion	0.136	0.021	0.044
Agreeableness	0.299^b^	0.329^b^	0.229^b^
Neuroticism	0.060	0.114	0.115

a *r* denotes the Pearson correlation coefficient.

b Values are statistically significant at *P*<.05. Full statistical results are reported in Table A3 of Multimedia Appendix 1.

As shown in Table 5, embedding-based LoRA models achieved stronger performance than other LoRA configurations. Conscientiousness achieved the highest correlation (*r*=0.352 at rank 8), which represents the best result within this model family. Openness and neuroticism also showed relatively stable and statistically significant performance across ranks (*r*≈0.23‐0.26).

**Table 5.** Performance of RoBERTa-base embedding-based low-rank adaptation (LoRA) fine-tuning across adapter ranks for predicting continuous Big Five personality scores from semistructured interviews in the pooled analytic sample (N=518).

Trait	Rank 8 (*r*^a^)	Rank 16 (*r*)	Rank 32 (*r*)
Openness	0.258^b^	0.251^b^	0.255^b^
Conscientiousness	0.352^b^	0.143	0.232^b^
Extraversion	0.147	0.143	0.197^b^
Agreeableness	0.184	0.172	0.173
Neuroticism	0.246^b^	0.232^b^	0.239^b^

a *r *denotes the Pearson correlation coefficient.

b Values are statistically significant at *P*<.05. Full statistical results are reported in Table A4 of Multimedia Appendix 1.

In contrast, extraversion and agreeableness were generally weaker, with only extraversion, at rank 32, reaching statistical significance. Increasing the LoRA rank did not consistently improve performance, as some traits achieved their best results at lower ranks. Overall, these findings suggest that embedding-based models captured some personality-relevant signals more consistently, although performance varied across traits.

### Supervised Fine-Tuning of GPT-4.1 mini

As shown in Table 6, fine-tuning results were largely consistent for conscientiousness, agreeableness, and neuroticism across the 2 runs. Conscientiousness showed the strongest performance (*r*=0.686 and 0.718), followed by agreeableness and neuroticism. Extraversion was less stable across runs (*r*=0.389 vs 0.172), while openness remained weak and nonsignificant (*r*=−0.085 and −0.138). These results suggest that fine-tuning more reliably captured certain personality traits, such as conscientiousness and agreeableness, while others remain more difficult to infer from text.

**Table 6.** Performance of GPT-4.1 mini supervised fine-tuning across 2 independent training runs for predicting continuous Big Five personality scores from semistructured interviews in the pooled analytic sample (N=518).

Trait	FT^a^1 (*r*^b^)	FT2 (*r*)
Openness	−0.085	−0.138
Conscientiousness	0.686^c^	0.718^c^
Extraversion	0.389^c^	0.172
Agreeableness	0.626^c^	0.631^c^
Neuroticism	0.422^c^	0.403^c^

a FT: fine-tuning.

b *r* denotes the Pearson correlation coefficient.

c Values are statistically significant at *P*<.05. Full statistical results are reported in Table A5 of Multimedia Appendix 1.

### Embedding-Based Regression

As shown in Table 7, embedding-based models achieved moderate, trait-dependent performance across pretrained encoders. Overall, text-embedding-3-large performed best, yielding the highest correlations for conscientiousness (*r*=0.568), agreeableness (*r*=0.531), and neuroticism (*r*=0.249), all of which are statistically significant.

**Table 7.** Performance of embedding-based regression using pretrained text encoders for predicting continuous Big Five personality scores from semistructured interviews in the pooled analytic sample (N=518).

Trait	all-mpnet-base-v2	E5-large	text-embedding-3-small	text-embedding-3-large
Openness	−0.098	−0.036	0.189	0.060
Conscientiousness	0.174	0.386^a^	0.476^a^	0.568^a^
Extraversion	0.302^a^	0.132	0.102	0.250^a^
Agreeableness	0.381^a^	0.407^a^	0.448^a^	0.531^a^
Neuroticism	0.040	0.096	0.119	0.249^a^

a Values are statistically significant at *P*<.05. Full statistical results are reported in Table A6 of Multimedia Appendix 1.

Across traits, conscientiousness and agreeableness were the most consistently well-predicted, with significant correlations across multiple encoders. Extraversion showed modest-to-moderate performance, with significant results for all-mpnet-base-v2 (*r*=0.302) and text-embedding-3-large (*r*=0.250), but weaker performance for other models. In contrast, neuroticism showed limited performance, reaching statistical significance only with the largest embedding model. Openness remained difficult to predict across all encoders, with weak and nonsignificant correlations, consistent with patterns observed in other methods.

To examine whether predictive performance improved with larger training samples, we conducted a sample-size sensitivity analysis using the text-embedding-3-small, embedding-based regression model as a representative embedding baseline. Increasing proportions of the training pool were used for model training (20%, 40%, 60%, 80%, and 100%; corresponding to n=82, 165, 248, 331, and 414), while evaluation was performed on the same held-out test set. As shown in Table A8 of Multimedia Appendix 1, increasing the training sample did not consistently improve predictive performance. Averaged across the 5 traits, Pearson correlations were 0.312, 0.326, 0.305, 0.300, and 0.267, respectively. At the trait level, conscientiousness and neuroticism generally decreased as the training sample increased, with their highest correlations observed at the 20% condition (*r*=0.581 and *r*=0.227, respectively), both higher than the corresponding full-training values. Agreeableness peaked at the 40% condition (*r*=0.546) and then declined. In contrast, openness increased modestly across the training fractions, while extraversion increased through the 80% condition before declining at 100%; however, extraversion correlations remained below 0.20 across all training conditions. This pattern may partly reflect the use of a fixed ridge regularization parameter without systematic hyperparameter tuning or a separate validation set.

## Discussion

### Principal Findings

Across all modeling paradigms, personality prediction performance exhibits 2 consistent patterns: strong dependence on model adaptation strategy and substantial variation across personality traits. Consistent with our prior work on these data [24], prompt-based approaches (zero-shot and CoT prompting) yield weak and unstable performance. In contrast, supervised learning or embedding-based regression demonstrate clear improvements by enabling models to learn from labeled data. In addition, prediction performance varies across the Big Five traits, suggesting that different personality traits may benefit from different modeling approaches.

First, prompt-based inference methods without task-specific adaptation (zero-shot and CoT prompting) showed weak and unstable performance in directly predicting continuous Big Five personality scores from semistructured interview transcripts. In contrast, Wright et al [30] reported moderate convergence between LLM-generated and self-reported personality trait scores based on spontaneous thought narratives and daily video diaries collected from 2 distinct samples. Compared with their unconstrained narrative data, our study evaluated personality prediction from semistructured interview transcripts, which may partly explain the lower zero-shot performance. In our benchmark, supervised approaches consistently outperformed prompt-based inference.

Second, performance varies systematically across traits. Conscientiousness and agreeableness are consistently the most predictable traits, achieving moderate-to-strong correlations across multiple methods, particularly under supervised fine-tuning (*r*=0.718 for conscientiousness and *r*=0.631 for agreeableness). Neuroticism shows moderate and relatively consistent performance, whereas extraversion is more variable across methods and runs. In contrast, openness shows the strongest and most consistent performance under embedding-based LoRA fine-tuning with RoBERTa-base (*r*=0.251‐0.258), while LLaMA-2-7B LoRA achieves modest but statistically significant performance (best *r*=0.216). Prompt-based methods, supervised fine-tuning, and embedding-based regression remain comparatively weak, suggesting that prediction performance for openness is highly method-dependent.

Finally, increasing model complexity does not uniformly improve performance. While supervised fine-tuning provides the strongest overall results, parameter-efficient methods such as LoRA yield only modest gains, and embedding-based approaches offer a competitive trade-off between performance and efficiency. The sample-size sensitivity analysis did not show a clear performance gain within the current training range; however, larger cohorts and systematic variation in transcript length are still needed to determine the data conditions required for stable personality estimates.

These results are consistent with prior work showing that narrative interview language can be used to predict Big Five personality scores when language models are trained on validated psychometric measures [79]. They also support recent findings that free-form language may require psychologically grounded supervision to connect linguistic cues with questionnaire-based personality constructs [80].

The stronger performance of supervised fine-tuning, compared with prompting and most parameter-efficient configurations, indicates that mapping language representations to validated personality constructs requires direct optimization. At the same time, the competitive performance of embedding-based regression highlights that meaningful personality signals are already present in pretrained representations but require appropriate extraction mechanisms. In our implementation, transcripts are segmented into fixed-length chunks (512 tokens) and aggregated using mean pooling, which may smooth localized linguistic signals in longer conversational inputs.

The observed trait-specific differences further suggest that not all personality dimensions are equally expressed in language. Traits such as conscientiousness and agreeableness appear to have stronger and more stable linguistic markers, whereas openness may depend on richer contextual or cognitive signals that are not fully captured by the available interview responses [81,82].

### Limitations and Future Work

Although this study provides a systematic comparison of 4 modeling paradigms for personality inference from interview transcripts, several limitations constrain the generalizability of our findings.

First, although the dataset is based on real-world interview data, the sample size (n=518) remains modest relative to the parameter scale of large models, particularly decoder-based architectures such as LLaMA-2-7B. The sample-size sensitivity analysis did not show a clear performance gain within the current training range; however, future work should examine larger cohorts and systematically vary both participant sample size and transcript length to determine the data conditions needed for stable personality estimates. In addition, because the analytic sample pooled participants across occupational, clinical, student, and community cohorts, the findings may not generalize uniformly to any single population.

Second, several limitations also apply specifically to the embedding-based regression models. Variability in transcript length and the use of truncation, chunking, or mean pooling may lead to semantic information loss when long interview responses are compressed into fixed-size representations [83]. The ridge regression models were trained with a fixed regularization setting, without systematic hyperparameter tuning or a separate validation set for model selection; therefore, these results should be interpreted as baseline comparisons rather than fully optimized estimates of embedding-based model performance. Model performance was also evaluated using a single 80 to 20 train-test split rather than repeated k-fold cross-validation, which may make the reported estimates sensitive to the specific hold-out partition. Future work should use repeated cross-validation or external validation to better assess the robustness of these findings.

Third, the use of the short-form BFI-10 introduces inherent psychometric limitations. In the present sample, Cronbach α ranged from 0.429 for openness to 0.693 for conscientiousness, indicating nontrivial measurement noise in the self-reported criterion scores, which may attenuate observed model-criterion correlations and contribute to the apparent performance ceiling. It is also important to clarify that the criterion variables in this study are self-reported Big Five trait scores rather than personality in its full conceptual scope. Self-reports capture one form of personality-related information, partly reflecting individuals’ explicit self-concepts and self-images [5]. Accordingly, LLM-derived estimates from interview language should be interpreted as predictions of self-reported trait scores, not as direct measurements of personality itself.

Fourth, our evaluation of prompting methods was limited to zero-shot and CoT strategies, which do not fully capture the broader space of prompt design strategies. Future studies should investigate few-shot prompting, trait-specific instructions, and prompting strategies that explicitly incorporate emotional or contextual signals, particularly for traits such as neuroticism.

Fifth, while we explored parameter-efficient adaptation using LoRA, our analysis was limited to variations in rank under a fixed configuration. Other factors, such as layer-wise adaptation, learning rate scheduling, and integration with retrieval-based methods (eg, RAG), may further improve performance, particularly for effectively salient traits such as neuroticism.

Finally, all models evaluated in this study were general-purpose pretrained models. Domain-adapted models trained on psychologically relevant or interview-based corpora may encode more informative representations for personality inference. Future work should compare language-based estimates with longer personality inventories, informant reports, behavioral indicators, and other criterion measures to evaluate whether they provide complementary information beyond self-report.

### Conclusions

This study shows that personality inference from natural language is both feasible and fundamentally constrained, revealing a pattern that is expressive but uneven and highly dependent on correspondence between language representations and psychometric constructs.

Contemporary LLMs encode meaningful personality-related signals but do not reliably translate these signals into calibrated trait estimates without explicit supervision. This pattern helps explain why models that appear linguistically fluent may nevertheless fail to produce stable or consistent personality predictions. Reliable personality inference therefore depends not only on model capability but also on how representations are aligned, adapted, and evaluated against validated psychological frameworks.

From a modeling perspective, personality prediction should be understood as a measurement problem rather than a purely generative task, in which performance is shaped by the interaction between data structure, model adaptation, and trait-specific linguistic expressivity. This reframing highlights that improving personality inference requires not only larger models but also a more principled integration of psychometric theory and representation learning.

Future work should extend this framework to more interactive and longitudinal settings, examining how personality signals evolve across extended conversations and diverse contexts. Additionally, incorporating richer personality measures and domain-adapted models may improve the reliability of trait-level inference.

## Supplementary material

10.2196/87129 Multimedia Appendix 1 Prompt templates, example model outputs, and supplementary statistical results.

## Abbreviations

BERT: bidirectional encoder representations from transformers
BFI-10: 10-item Big Five Inventory
CoT: chain-of-thought
LLaMA: Large Language Model Meta AI
LLM: large language model
LoRA: low-rank adaptation
MAE: mean absolute error
OSF: Open Science Framework
RAG: retrieval-augmented generation
RoBERTa: Robustly Optimized BERT Approach
