# Supplementary material for: Personality Inference From Semistructured Interviews Using Large Language Models: Comparative Evaluation Study
Source: J Med Internet Res. 2026 Oct 16;28:e87129. doi: 10.2196/87129 (PMC13674523; doi:10.2196/87129)
Supplement: Supplementary file 1 [file jmir-v28-e87129-s001.docx]

**Multimedia Appendix 1. Prompt templates, example model outputs, and supplementary statistical results.**

1. **Prompt Templates and Interview Prompts**

**Table A1. Semi-structured interview prompts used across the pooled parent studies.**

| **Parent Study** | **Population** | **Baseline** | **Q1** | **Q2** | **Q3/QV** | **Q4** |
| --- | --- | --- | --- | --- | --- | --- |
| Akron Fire Department Resilience Project | Active-duty firefighters | Previous-day activities | Experience of being a firefighter | Coping with firefighter work | Scary/frightening event | Positive firefighter event |
| Adjustment to Multiple Sclerosis Project | MS patients | Previous-day activities | Experience of having MS | Coping with MS | Negative mood-inducing video | Recent positive event |
| Resilience and Adaptation to Injury Project | Post-PTSD-event adults | Previous-day activities | Describe Traumatic Event and Injury | Coping since injury | Recent negative event | Recent positive event |
| Transition to College Project | First-semester students | Previous-day activities | Difficult/distressing college event | Positive/happy college event | — | — |
| Emotion Attention and Mood in Daily Life | Community adults | Previous-day activities | Negative autobiographical event | Positive autobiographical event | Event experience rejection | Event experience support |

T**extbox 1. Zero-Shot Prompt used for Big Five personality prediction.**

A zero-shot prompting strategy was used to directly infer personality trait scores from interview transcripts without intermediate reasoning. The model was instructed to output continuous Big Five scores based solely on observable linguistic and behavioral patterns.

"""You are a psychology expert trained in personality assessment using the Big Five model (Five-Factor Model, FFM).

Task:

Based on the following interview transcript, estimate the individual's Big Five personality traits.

Instructions:

- Infer each trait based on observable behavioral, emotional, and linguistic patterns in the text.

- Use the full range of the scale.

- Scores must be continuous values between 1.0 (very low) and 5.0 (very high).

- Do not round to integers unless strongly justified.

Return ONLY in this exact format (no extra text, no explanation):

Openness: X.X

Conscientiousness: X.X

Extraversion: X.X

Agreeableness: X.X

Neuroticism: X.X

{text}

"""

**Textbox 2. Chain-of-Thought Prompt used for Big Five personality prediction.**

A chain-of-thought (CoT) prompting strategy was employed to encourage structured reasoning prior to score assignment. The model was guided to (1) extract behavioral evidence, (2) reason about each trait, and (3) assign continuous scores, thereby improving interpretability of the prediction process.

"""

You are a psychology expert trained in personality assessment using the Big Five model (Five-Factor Model, FFM).

Task:

Based on the following interview transcript, estimate the individual's Big Five personality traits using a step-by-step reasoning process.

Instructions:

Step 1 - Extract behavioral evidence:

Identify and list specific behavioral, emotional, and linguistic cues from the transcript that are relevant to each trait. Quote or paraphrase directly from the text.

Step 2 - Reason about each trait:

For each of the five traits, reason explicitly about whether the evidence supports a high, moderate, or low score. Consider both presence and absence of relevant cues.

Step 3 - Assign scores:

Based on your reasoning, assign a continuous score between 1.0 (very low) and 5.0 (very high) for each trait. Use the full range of the scale. Do not round to integers unless strongly justified.

Important:

- Base your judgment only on evidence from the text.

- Do not assume traits without textual support.

- Be consistent across traits.

- Think carefully before assigning each score.

Now analyze the following transcript:

{text}

First, work through Steps 1-3 above. Then, at the end, output ONLY the final scores in this exact format (no extra text after the scores):

Openness: X.X

Conscientiousness: X.X

Extraversion: X.X

Agreeableness: X.X

Neuroticism: X.X

"""

**2. Example Model Output**

**Textbox 3.** Example of GPT-5 Mini chain-of-thought reasoning for personality trait inference from a representative interview transcript.

**Step 1 — Extract behavioral evidence (quotes/paraphrase tied to each trait)**

Openness

- “it’s ….” (expresses appreciation for variety in work)

- “I’m …” (interest in further learning)

- Describes training, ….

- Hobbies described: ….

Conscientiousness

- Detailed list of chores: ….

- Responsibility at work: ….

- Planning/rest routines: ….

- Took initiative for colleagues: ….

Extraversion

- Social affiliation: …

- Enjoys public-facing activities: …

- Social/leisure: …

- Some solo preferences: …

Agreeableness

- Prosocial acts and empathy: …

- Values helping people and public education; also expresses moral negativity toward system abusers: …

- Comments about workplace gossip: …

Neuroticism

- Acute negative reactions in stressful events: scared by dog incident….

- Emotional recovery and coping: ….

- No pervasive self-focused anxiety or ruminative language; mostly situational stress with effective coping.

**Step 2 — Reasoning about each trait (high/moderate/low)**

Openness — Moderate

- Evidence shows willingness to learn (…), curiosity about varied work tasks, and tolerance for novelty (different calls, specialties). However, hobbies are practical (…) rather than strongly artistic or highly imaginative. Overall supports a moderate level of openness.

Conscientiousness — High

- Repeated concrete examples of routine maintenance, cleanliness, preparation, duty fulfillment, training upkeep, and responsibility toward colleagues and patients indicate strong organization, reliability, and discipline. Supports a high score.

Extraversion — Moderate–High

- Strong social orientation toward team, enjoyment of public-facing tasks, and frequent social leisure indicate higher-than-average sociability and positive engagement. Presence of solitary coping activities tempers a maximal extraversion rating. Supports moderate–high.

Agreeableness — High

- Frequent prosocial behaviors, empathy, helping others, and personal gestures (taking brother out, comforting patients) indicate high compassion and cooperativeness. Frustration with system abusers and recognition of gossip reduce nothing substantial; overall pattern is agreeable.

Neuroticism — Low–Moderate (leaning low)

- The individual experiences situational fear and worry in dangerous calls but demonstrates effective coping, quick recovery, and pride in successes. No signs of chronic anxiety, moodiness, or emotional instability. Supports a low-to-moderate score, closer to low.

**Step 3 — Assign continuous scores (1.0 very low — 5.0 very high)**

Openness: 3.2

Conscientiousness: 4.4

Extraversion: 4.0

Agreeableness: 4.1

Neuroticism: 2.4

**3. Supplementary Tables**

**Table A2.** Full statistical results for GPT-5 Mini prompt-based Big Five personality prediction.

| **Trait** | **Method** | **r** | **p-value** | **CI Lower** | **CI Upper** | **MAE** |
| --- | --- | --- | --- | --- | --- | --- |
| Openness | Zero-shot | -0.034 | 0.441 | -0.120 | 0.052 | 0.714 |
|  | CoT | -0.010 | 0.825 | -0.096 | 0.077 | 0.695 |
| Conscientiousness | Zero-shot | 0.158 | **0.0003** | 0.072 | 0.240 | 1.458 |
|  | CoT | 0.155 | **0.0004** | 0.070 | 0.238 | 1.441 |
| Extraversion | Zero-shot | -0.092 | **0.037** | -0.176 | -0.005 | 1.189 |
|  | CoT | -0.092 | **0.037** | -0.177 | -0.006 | 1.146 |
| Agreeableness | Zero-shot | 0.067 | 0.130 | -0.020 | 0.152 | 1.682 |
|  | CoT | 0.099 | **0.025** | 0.013 | 0.183 | 1.581 |
| Neuroticism | Zero-shot | -0.026 | 0.560 | -0.112 | 0.061 | 1.269 |
|  | CoT | -0.034 | 0.443 | -0.120 | 0.053 | 1.286 |

**Table A3.** Full statistical results for Big Five personality prediction using LLaMA-2-7B LoRA fine-tuning.

| **Trait** | **Rank** | **r** | **p-value** | **CI Lower** | **CI Upper** |
| --- | --- | --- | --- | --- | --- |
| Openness | 8 | 0.042 | 0.669 | -0.151 | 0.233 |
|  | 16 | 0.127 | 0.198 | -0.067 | 0.312 |
|  | 32 | 0.216 | **0.028** | 0.024 | 0.392 |
| Conscientiousness | 8 | -0.057 | 0.567 | -0.247 | 0.137 |
|  | 16 | 0.032 | 0.747 | -0.162 | 0.223 |
|  | 32 | -0.055 | 0.578 | -0.245 | 0.139 |
| Extraversion | 8 | 0.136 | 0.170 | -0.059 | 0.320 |
|  | 16 | 0.021 | 0.834 | -0.172 | 0.213 |
|  | 32 | 0.044 | 0.659 | -0.150 | 0.234 |
| Agreeableness | 8 | 0.299 | **0.002** | 0.113 | 0.465 |
|  | 16 | 0.329 | **0.0007** | 0.146 | 0.490 |
|  | 32 | 0.229 | **0.020** | 0.038 | 0.404 |
| Neuroticism | 8 | 0.060 | 0.543 | -0.134 | 0.250 |
|  | 16 | 0.114 | 0.248 | -0.080 | 0.300 |
|  | 32 | 0.115 | 0.244 | -0.079 | 0.301 |

**Table A4.** Full statistical results for Big Five personality prediction using RoBERTa-base with embedding-based LoRA fine-tuning across adapter ranks.

| **Trait** | **Rank** | **r** | **p-value** | **CI Lower** | **CI Upper** | **MAE** |
| --- | --- | --- | --- | --- | --- | --- |
| Extraversion | 8 | 0.147 | 0.144 | -0.051 | 0.334 | 0.947 |
|  | 16 | 0.1432 | 0.155 | -0.0548 | 0.3303 | 0.9504 |
|  | 32 | 0.197 | **0.0496** | 0.001 | 0.379 | 0.930 |
| Agreeableness | 8 | 0.184 | 0.068 | -0.013 | 0.367 | 0.694 |
|  | 16 | 0.172 | 0.086 | -0.025 | 0.357 | 0.731 |
|  | 32 | 0.173 | 0.084 | -0.024 | 0.358 | 0.738 |
| Conscientiousness | 8 | 0.352 | **0.0003** | 0.168 | 0.513 | 0.778 |
|  | 16 | 0.1429 | 0.156 | -0.0551 | 0.330 | 0.752 |
|  | 32 | 0.232 | **0.020** | 0.039 | 0.411 | 0.691 |
| Neuroticism | 8 | 0.246 | 0.035 | 0.016 | 0.392 | 0.962 |
|  | 16 | 0.232 | **0.020** | 0.038 | 0.410 | 0.999 |
|  | 32 | 0.239 | **0.017** | 0.044 | 0.416 | 0.958 |
| Openness | 8 | 0.258 | **0.010** | 0.065 | 0.432 | 0.431 |
|  | 16 | 0.251 | **0.012** | 0.057 | 0.426 | 0.500 |
|  | 32 | 0.255 | **0.011** | 0.061 | 0.430 | 0.499 |

**Table A5.** Full statistical results for Big Five personality prediction using GPT-4.1 Mini supervised fine-tuning across two independent runs.

| **Trait** | **Method** | **Pearson r** | **p-value** | **CI Lower** | **CI Upper** | **MAE** |
| --- | --- | --- | --- | --- | --- | --- |
| Openness | FT1 | -0.085 | 0.392 | -0.273 | 0.110 | 0.529 |
|  | FT2 | -0.138 | 0.161 | -0.322 | 0.056 | 0.563 |
| Conscientiousness | FT1 | 0.686 | **< 0.001** | 0.569 | 0.776 | 0.793 |
|  | FT2 | 0.718 | **< 0.001** | 0.609 | 0.800 | 0.721 |
| Extraversion | FT1 | 0.389 | **< 0.001** | 0.212 | 0.541 | 0.913 |
|  | FT2 | 0.172 | 0.082 | -0.022 | 0.352 | 1.010 |
| Agreeableness | FT1 | 0.626 | **< 0.001** | 0.493 | 0.730 | 0.750 |
|  | FT2 | 0.631 | **< 0.001** | 0.499 | 0.734 | 0.736 |
| Neuroticism | FT1 | 0.422 | **< 0.001** | 0.250 | 0.569 | 1.149 |
|  | FT2 | 0.403 | **< 0.001** | 0.228 | 0.553 | 1.087 |

**Table A6.** Full statistical results for Big Five personality prediction using embedding-based Ridge regression across pretrained text encoders.

| **Trait** | **Model** | **r** | **p-value** | **CI Lower** | **CI Upper** | **MAE** |
| --- | --- | --- | --- | --- | --- | --- |
| Openness | all-mpnet-base-v2 | -0.098 | 0.324 | -0.285 | 0.097 | 0.911 |
|  | E5-large | -0.036 | 0.716 | -0.227 | 0.158 | 0.749 |
|  | text-embedding-3-small | 0.189 | 0.055 | -0.004 | 0.368 | 0.643 |
|  | text-embedding-3-large | 0.060 | 0.546 | -0.134 | 0.250 | 0.628 |
| Conscientiousness | all-mpnet-base-v2 | 0.174 | 0.077 | -0.019 | 0.355 | 1.395 |
|  | E5-large | 0.386 | <0.001 | 0.209 | 0.539 | 1.151 |
|  | text-embedding-3-small | 0.476 | <0.001 | 0.312 | 0.612 | 0.997 |
|  | text-embedding-3-large | 0.568 | <0.001 | 0.421 | 0.685 | 0.917 |
| Extraversion | all-mpnet-base-v2 | 0.302 | 0.002 | 0.116 | 0.467 | 1.134 |
|  | E5-large | 0.132 | 0.180 | -0.062 | 0.317 | 1.194 |
|  | text-embedding-3-small | 0.102 | 0.305 | -0.093 | 0.288 | 1.121 |
|  | text-embedding-3-large | 0.250 | 0.010 | 0.061 | 0.423 | 0.985 |
| Agreeableness | all-mpnet-base-v2 | 0.381 | <0.001 | 0.204 | 0.535 | 1.073 |
|  | E5-large | 0.407 | <0.001 | 0.232 | 0.556 | 0.970 |
|  | text-embedding-3-small | 0.448 | <0.001 | 0.279 | 0.589 | 0.946 |
|  | text-embedding-3-large | 0.531 | <0.001 | 0.377 | 0.656 | 0.790 |
| Neuroticism | all-mpnet-base-v2 | 0.040 | 0.688 | -0.154 | 0.231 | 1.473 |
|  | E5-large | 0.096 | 0.332 | -0.098 | 0.283 | 1.335 |
|  | text-embedding-3-small | 0.119 | 0.230 | -0.076 | 0.304 | 1.262 |
|  | text-embedding-3-large | 0.249 | 0.011 | 0.060 | 0.422 | 1.152 |

**Table A7.** Descriptive statistics of BFI-10 Big Five trait scores in the analytic sample.

| Trait | Mean | Std | Skewness |
| --- | --- | --- | --- |
| Extraversion | 3.03 | 1.09 | -0.08 |
| Agreeableness | 2.63 | 1.13 | 0.30 |
| Conscientiousness | 2.74 | 1.30 | 0.43 |
| Neuroticism | 2.93 | 1.23 | 0.14 |
| Openness | 3.35 | 0.63 | 0.12 |

Descriptive statistics of Big Five personality scores derived from BFI-10 self-report measures. Values are reported on a 1–5 Likert scale. Mean and standard deviation summarize central tendency and variability, while skewness reflects distribution asymmetry across participants (N = 518).

**Table A8.** Sample-size sensitivity analysis using text-embedding-3-small embedding-based Ridge regression.

| Trait | Training fraction | Training N | Mean Pearson *r* | SD *r* | Mean MAE | SD MAE | Mean RMSE | SD RMSE |
| --- | --- | --- | --- | --- | --- | --- | --- | --- |
| Agreeableness | 20% | 82 | 0.528 | 0.060 | 0.802 | 0.050 | 0.982 | 0.060 |
|  | 40% | 165 | **0.546** | 0.023 | 0.792 | 0.022 | 0.983 | 0.029 |
|  | 60% | 248 | 0.537 | 0.048 | 0.812 | 0.045 | 1.000 | 0.056 |
|  | 80% | 331 | 0.499 | 0.054 | 0.853 | 0.053 | 1.047 | 0.053 |
|  | 100% | 414 | 0.449 | 0.000 | 0.945 | 0.000 | 1.163 | 0.000 |
| Conscientiousness | 20% | 82 | **0.581** | 0.075 | 0.881 | 0.079 | 1.087 | 0.090 |
|  | 40% | 165 | 0.568 | 0.075 | 0.881 | 0.092 | 1.119 | 0.087 |
|  | 60% | 248 | 0.523 | 0.053 | 0.919 | 0.081 | 1.180 | 0.063 |
|  | 80% | 331 | 0.512 | 0.041 | 0.946 | 0.062 | 1.219 | 0.048 |
|  | 100% | 414 | 0.477 | 0.000 | 0.996 | 0.000 | 1.267 | 0.000 |
| Extraversion | 20% | 82 | 0.082 | 0.050 | 1.013 | 0.028 | 1.249 | 0.045 |
|  | 40% | 165 | 0.133 | 0.087 | 1.027 | 0.065 | 1.276 | 0.084 |
|  | 60% | 248 | 0.137 | 0.081 | 1.053 | 0.100 | 1.318 | 0.101 |
|  | 80% | 331 | **0.143** | 0.078 | 1.067 | 0.032 | 1.344 | 0.047 |
|  | 100% | 414 | 0.102 | 0.000 | 1.124 | 0.000 | 1.407 | 0.000 |
| Neuroticism | 20% | 82 | **0.227** | 0.045 | 1.045 | 0.013 | 1.290 | 0.043 |
|  | 40% | 165 | 0.217 | 0.126 | 1.112 | 0.123 | 1.368 | 0.122 |
|  | 60% | 248 | 0.151 | 0.044 | 1.174 | 0.054 | 1.461 | 0.039 |
|  | 80% | 331 | 0.160 | 0.077 | 1.203 | 0.065 | 1.509 | 0.083 |
|  | 100% | 414 | 0.116 | 0.000 | 1.263 | 0.000 | 1.605 | 0.000 |
| Openness | 20% | 82 | 0.141 | 0.100 | 0.535 | 0.047 | 0.686 | 0.049 |
|  | 40% | 165 | 0.166 | 0.066 | 0.538 | 0.018 | 0.708 | 0.022 |
|  | 60% | 248 | 0.176 | 0.102 | 0.592 | 0.021 | 0.761 | 0.040 |
|  | 80% | 331 | 0.185 | 0.032 | 0.626 | 0.018 | 0.788 | 0.027 |
|  | 100% | 414 | **0.189** | 0.000 | 0.643 | 0.000 | 0.808 | 0.000 |

Note: Training fractions refer to proportions of the 80% training pool. Performance was evaluated on the same held-out 20% test set. Values are averaged across five repeated subsamples for 20%–80%; the 100% condition uses the full training pool, so SD values are 0.000. MAE = mean absolute error; RMSE = root mean squared error. The 100% condition and the corresponding text-embedding-3-small results reported in the main text were obtained from separate evaluation runs using the same model configuration; minor numerical differences were observed between the stored outputs.

**Table A9.** Internal consistency of BFI-10 trait scales in the analytic sample.

| Trait | N complete | Cronbach’s alpha | Inter-item r | Inter-item p |
| --- | --- | --- | --- | --- |
| Extraversion | 518 | 0.579 | 0.408 | < .001 |
| Agreeableness | 518 | 0.578 | 0.410 | < .001 |
| Conscientiousness | 518 | 0.693 | 0.531 | < .001 |
| Neuroticism | 518 | 0.674 | 0.510 | < .001 |
| Openness | 518 | 0.429 | 0.278 | < .001 |

**Note:** Internal consistency was calculated using the item scoring direction used to derive the final BFI-10 trait scores. Because each BFI-10 trait is represented by two items, inter-item correlations are also reported.
